# The passive surveillance of ticks using companion animal electronic health records

**DOI:** 10.1017/S0950268817000826

**Published:** 2017-05-02

**Authors:** J. S. P. TULLOCH, L. MCGINLEY, F. SÁNCHEZ-VIZCAÍNO, J. M. MEDLOCK, A. D. RADFORD

**Affiliations:** 1 NIHR Health Protection Research Unit in Emerging and Zoonotic Infections, University of Liverpool, Liverpool, UK; 2 Institute of Infection and Global Health, The Farr Institute@HeRC, University of Liverpool, Waterhouse Building (2nd Floor, Block F), 1-5 Brownlow Street, Liverpool, L69 3GL, UK; 3 Medical Entomology Group, Emergency Response Department, Public Health England, Porton Down, Salisbury, SP4 0JG, UK; 4 NIHR Health Protection Research Unit in Health and the Environment, Porton Down, SP4 0JG, UK; 5 Institute of Infection and Global Health, University of Liverpool, Leahurst Campus, Chester High Road, Neston, S. Wirral, CH64 7TE, UK

**Keywords:** Companion animals, electronic health records, Great Britain, one health, surveillance, ticks

## Abstract

Ticks represent a large global reservoir of zoonotic disease. Current surveillance systems can be time and labour intensive. We propose that the passive surveillance of companion animal electronic health records (EHRs) could provide a novel methodology for describing temporal and spatial tick activity. A total of 16 58 857 EHRs were collected over a 2-year period (31 March 2014 and 29 May 2016) from companion animals attending a large sentinel network of 192 veterinary clinics across Great Britain (the Small Animal Veterinary Surveillance Network – SAVSNET). In total, 2180 EHRs were identified where a tick was recorded on an animal. The relative risk of dogs presenting with a tick compared with cats was 0·73 (95% confidence intervals 0·67–0·80). The highest number of tick records were in the south central regions of England. The presence of ticks showed marked seasonality with summer peaks, and a secondary smaller peak in autumn for cats; ticks were still being found throughout most of Great Britain during the winter. This suggests that passive surveillance of companion animal EHRs can describe tick activity temporally and spatially in a large cohort of veterinary clinics across Great Britain. These results and methodology could help inform veterinary and public health messages as well as increase awareness of ticks and tick-borne diseases in the general population.

## INTRODUCTION

Ticks are effective vectors of zoonotic pathogens, and tick-borne diseases (TBDs) can be severely debilitating to both humans and companion animals, in some cases leading to death. Lyme disease is the most common TBD in the Northern Hemisphere with, in Western Europe, an unweighted mean for annual incidence rate of 56·3/1 00 000 persons per year [1]. TBDs can pose a large burden on health services; a recent study of Lyme borreliosis inpatients in Germany estimated an annual cost in excess of 30 million Euros [2]. Due to this, and increasing public concern, governments and research organisations are trying to heighten and improve their understanding of risk models of ticks [3]. The responses to this call have largely fallen into three categories: the active and passive collection of ticks, the utilisation of digital applications, and the monitoring of electronic health records (EHRs).

In Great Britain (GB), Public Health England (PHE) coordinates the Tick Surveillance Scheme (TSS) [4, 5], which relies on passive submission of ticks by members of the public as well as medical/veterinary professionals. Between 2005 and 2016 the TSS received a total of 18 000 ticks, primarily found on companion animals and humans (PHE unpublished). Similarly, ‘the Big Tick Project’, collected data on ticks found on dogs in the UK [6, 7]; in their most recent study, 6555 ticks were actively collected from dogs attending select veterinary clinics over a 16-week period from April to July 2015 [6]. Since such systems collect the actual tick, they are able to both identify the ticks and describe their spatial distributions. However, they are labour and time intensive, relying on large amounts of public engagement and involvement, and in the case of the Big Tick Project, do not provide continuous surveillance data.

A very different approach has been developed by the National Institute for Public Health and the Environment (RIVM) in the Netherlands. The Tekenradar website and digital app allows members of the public to record when they have been bitten by a tick (and send it to RIVM), or develop an erythema migrans rash which is pathognomonic for Lyme disease [8, 9]. This enables ‘live’ reporting of tick bites and identifies areas of tick bite and Lyme disease risk. Due to its presence on multiple digital platforms it facilitates easy promotion for public health messaging. It has also been promoted as a resource for researchers of ticks and TBDs [9]. However, the success of such a system is largely reliant on the accurate diagnosis and identification of ticks, tick bites and erythema migrans by members of the public, rather than qualified health care professionals.

In Switzerland, the government has set up a voluntary surveillance system of 150 primary care physicians called Sentinella [10], recording 1644 cases of tick bites from 2008 to the end of 2011. Rather than collecting and submitting ticks for further analyses, this system relies on the accurate diagnosis and recording of tick bites by medical practitioners within their patients’ EHRs, without the actual visualisation or collection of the tick. In a similar way, PHE use routine passive syndromic surveillance based on a predetermined list of clinical codes to monitor the incidence of arthropod bites in near real-time across various clinical settings including general practitioner consultations, emergency department attendance and telephone helplines [11]. However, constraints of the clinical diagnostic codes being used mean tick bites cannot be analysed separately from those of other arthropods.

While each of these systems contribute to different aspects of tick surveillance, none of them currently provide a surveillance system that is low cost and in sufficient temporal and spatial resolution to quickly and efficiently provide large sets of data about generic tick activity.

According to the most recent estimates, there are 11·6 million dogs and 10·1 million cats kept as pets in the UK, with 30% and 23% of households owning a dog and cat, respectively [12]. These species have the potential for greater exposure to tick habitats than humans, and often without measures to prevent tick contact. It has been shown that dogs that are regularly walked are likely to acquire ticks, and it is well established that dogs have the potential to act as sentinels for ticks and TBDs [6, 13–16]. Due to owner concern, companion animals with ticks are often presented to veterinary clinics, with the veterinary practitioner frequently recording the presence of ticks within an individual animal's EHR [17]. The aim of this paper is to explore the feasibility of using such EHRs from a large sentinel network of veterinary clinics as the basis of a novel surveillance system to provide efficient temporal and spatial estimates of tick activity risk in GB that complement existing TSS.

## METHODS

**Fig. 1.**
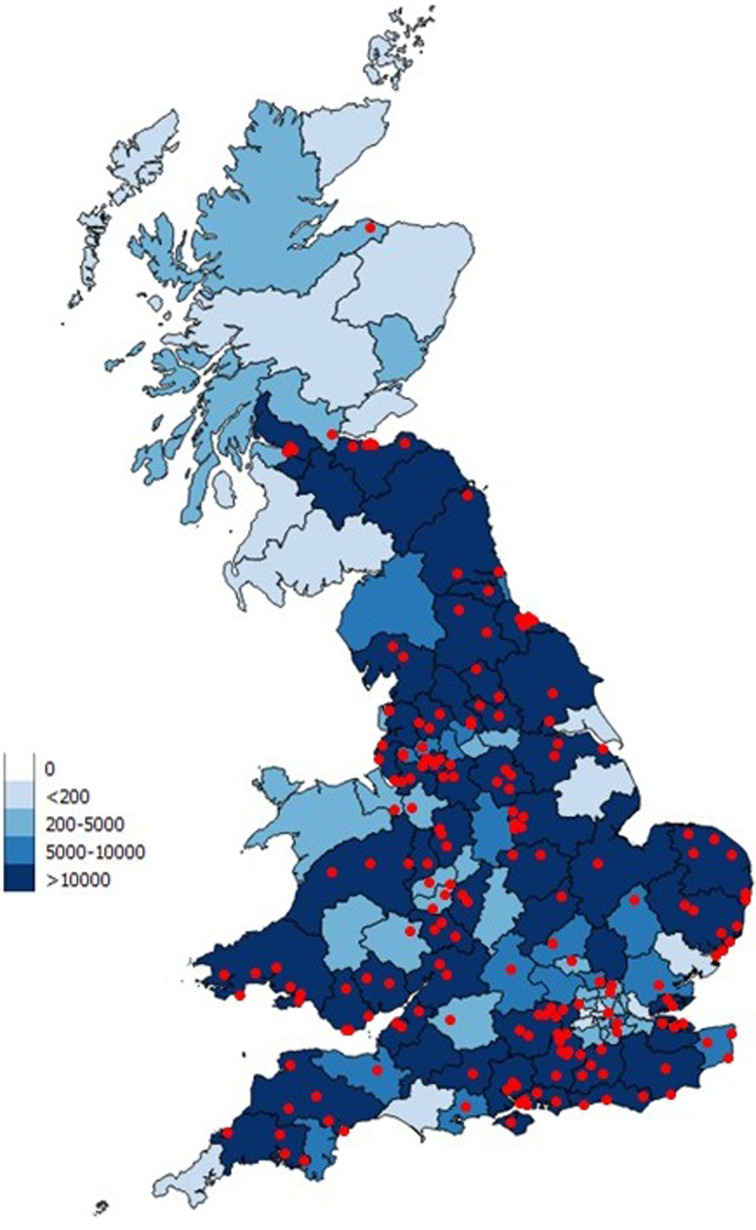
The distribution of participating SAVSNET veterinary clinics (red dots) within GB, and the total number of EHRs collected between April 2014 and May 2016 by owners’ postcode area.

EHRs were collected through the Small Animal Veterinary Surveillance Network (SAVSNET) from volunteer veterinary clinics using a compatible practice management system; currently Teleos™ and RoboVet™. This study uses over 2 years of data gathered from 192 veterinary clinics across the UK between 31 March 2014 and 29 May 2016 (Fig. 1). Each EHR was collected at the end of a veterinary consultation in real-time and included the following data; date of the consultation, postcode of the owner, species of the animal in the consultation and the clinical narrative, which would have been written by the consulting veterinary surgeon or nurse. Whilst data on ectoparasiticide treatments were collected, they were not included in further analysis; many are active against multiple arthropods and routine prophylactic prescription by veterinarians results in a low specificity for tick infestation.

Initially, a simple free-text analysis approach was developed to identify EHRs containing the word ‘tick’ in the clinical narrative field, whilst excluding records where only the terms ‘tickl’, ‘ticki’ and ‘sticki’ were used. To increase the specificity of such an approach, the resulting EHRs were subsequently read by two domain experts (J.S.P.T. and L.M.) who verified the reference to ticks based on a strict case definition. A tick was only deemed present in a consultation if, within the associated EHR, ‘a veterinary surgeon or nurse confirmed visual sighting or removal of a tick within the consultation.’ This case definition was used to avoid any misidentification errors of ticks by owners, or any historical identification of ticks being included in the current analysis (e.g. tick removed last week). To verify concordant interpretation of the case definition, the two domain experts manually classified a random sample of the EHRs. The amount of agreement (i.e. inter-rate reliability) was measured by Cohen's *κ* coefficient (R package ‘psych’) [18]. Each EHR where an agreement was not achieved was re-examined by both domain experts to develop a more consistent interpretation of the case definition. This process was repeated until an ‘almost perfect’ level of agreement was achieved, at which point the remaining EHRs were randomly divided between the two domain experts and categorised [19].

Results of these analyses were used to calculate the number of consultations where a tick was recorded in the EHR per 10 000 consultations. We assume this to be a proxy for activity of ticks and for brevity, refer to this measure as ‘tick activity’. Relative risks were calculated between the two predominant host species (i.e. dogs and cats) with statistical significance (*P* < 0·05) measured by a *χ*^2^ test.

Time series plots (based on the time of the consultation as recorded in the EHR) were used to identify temporal trends in tick activity and to compare temporal trends by host species. The temporal pattern of tick activity was smoothed using a non-parametric method, the LOESS (locally weighted regression) technique (R package ‘ggplot2’) [20, 21]. Outliers were identified as data points outside the smoothed data's 95% confidence intervals (CIs). All proportions and 95% CIs were calculated using robust standard errors to account for intragroup correlation within veterinary clinics. Statistical analyses were carried out using R language (version 3.2.0) (R Core Team 2015).

Maps were used to describe the spatial distribution of tick activity during each season. We defined season as winter (December–February), spring (March–May), summer (June–August) and autumn (September–November). The spatial distribution of tick activity was stratified by owner's given address. SAVSNET receives full owner postcode for each EHR, which locates each address to one of 1·75 million locations [22]. However at such resolution it is possible to identify some individual properties, particularly in rural areas. Therefore, postcode area (first half of postcode; *n* = 124) was used to maintain owner confidentiality when presenting the results. When displaying the data, we took a cautious approach and excluded areas with <200 EHRs in each season, as they were less likely to be representative. A map was constructed displaying all EHRs, aggregated by owner's postcode area, to show the underlying population distribution (Fig. 1). The data was depicted using QGIS version 2.8.2-Wien.

## RESULTS

In total 16 58 857 EHRs were collected during the study period, consisting of 70·5% dogs and 26·4% cats. Of these, 10 155 (0·61%) had a clinical narrative containing the word ‘tick’. The two domain experts first independently read and applied the case definition to 365 randomly selected EHRs from these 10 155. After adjusting by the amount of agreement which would be expected by chance, a ‘substantial agreement’ (*K* = 0·7; 95% CI 0·63–0·78) with 305 EHRs agreed was achieved. After reappraising this first dataset, the exercise was repeated on a new random sample of 365 EHRs, this time achieving an ‘almost perfect’ agreement (*K* = 0·82; 95% CI 0·77–0·88) with 332 EHRs agreed; the remaining EHRs were therefore categorised independently by the two authors.

In total, 2180 EHRs were confirmed as having a tick present, equating to 0·13% of the total 16 58 857, and 21·5% of the 10 155 automatically identified EHRs. Of these 2180 EHRs, 1421 were from dogs (65·2%), 728 from cats (33·4%), and 17 from other species (which only included ferrets, rabbits and guinea pigs; 0·8%), with the remaining 14 EHRs lacking an identifiable species label (0·6%). The relative risk of a dog being recorded as presenting with a tick compared with that for a cat was 0·73 (95% CI 0·67–0·80, *P* < 0·005). The main reasons for EHRs being identified by the free-text analysis but failing to meet the case definition included; misidentification of ticks by owners (e.g. skin tags, nipples, tumours), ticks observed by owners before the consultation and not confirmed by a veterinary surgeon or nurse within the consultation, and discussions held in the consultation about ticks and TBDs without a tick being present. Only five of the 2180 (0·2%) EHRs identified as relating to ticks included information at genus and species level; two referring to *Ixodes* spp, one to *I. ricinus*, one to *Dermacentor* spp and one EHR referring to both *Dermacentor and Rhipicephalus* spp.

**Fig. 2.**
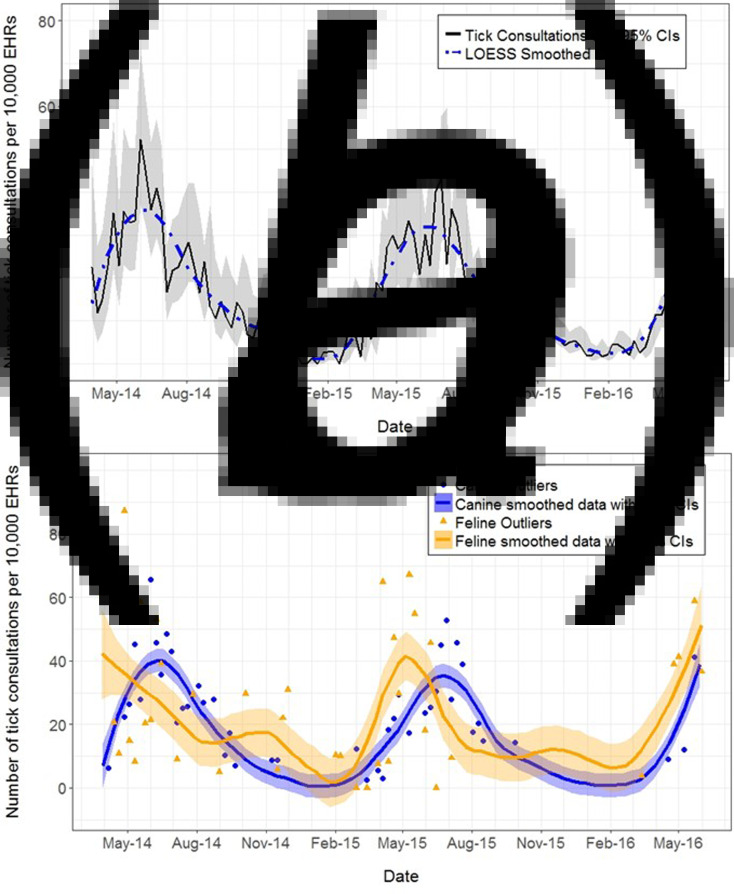
Time series plot showing the weekly number of tick-based EHRs per 10 000 EHRs (a) between April 2014 and May 2016, in GB; and (b) in dogs and cats between April 2014 and May 2016, in GB.

The mean weekly rate of tick reporting in this population over the entire study period was 15·3 tick-based EHRs per 10 000 EHRs. The temporal pattern was similar in both calendar years, with peak tick activity between May and July each year, and highest levels recorded in mid-June (Fig. 2a). Minimum tick activity was between December and February, with the lowest activity in January in both calendar years. The temporal pattern of tick activity in dogs was similar to the overall population, with peak activity in June (maximum of 65·5 tick-based EHRs per 10 000 EHRs over a single week) and lowest levels between December and February (Fig. 2b). In contrast, cats seemed to have an earlier peak in weekly tick activity in May (with a maximum of 87·2 tick-based EHRs per 10 000 EHRs), with a secondary smaller peak in the autumn, and their lowest levels in February (Fig. 2b). In the winter of 2015–2016, ticks were still recorded in every week on cats, whilst for two separate weeks none were recorded on dogs. The mean weekly rate of tick activity was lower for dogs (14·8 tick-based EHRs per 10 000 EHRs) than for cats (18·3 tick-based EHRs per 10 000 EHRs).

**Fig. 3.**
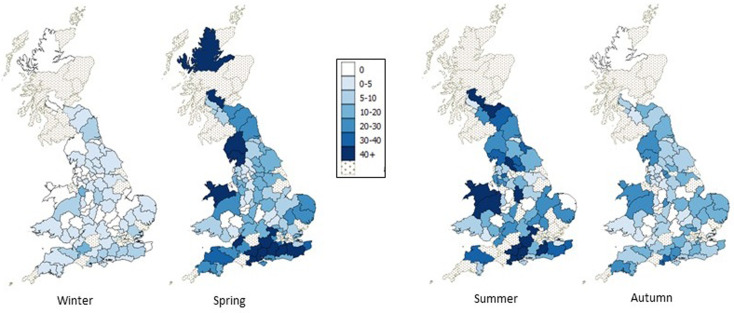
Geographical distribution of tick-based EHRs per 10 000 EHRs in GB, aggregated by owners’ postcode area for each season between April 2014 and May 2016. The dotted postcode areas represent areas with <200 EHRs in total during the relevant time period.

There was considerable variation in the spatial distribution of tick activity across each postcode area in GB (Fig. 3). The 10 postcode areas with highest tick activity across all seasons were (in descending numerical order); Bournemouth, Hemel Hempstead, Southampton, Falkirk, Salisbury, Guildford, Croydon, Llandudno, Reading and Lancaster. Of these, Southampton, Bournemouth, Guildford, Reading, Llandudno and Lancaster peaked in spring; the remainder peaking in summer. No postcode areas had their peak activity in autumn or winter. The areas with no tick activity across all seasons were Hereford, Oldham and Wolverhampton.

## DISCUSSION

Ticks are an important vector of disease but continuous surveillance has proved challenging. Here we show how text mining of companion animal EHRs from a large sentinel population of veterinary clinics may provide a novel form of passive surveillance to describe temporal and spatial trends in tick activity across GB. This could inform more targeted public health messaging to the risk of veterinary disease and human health risks associated with ticks.

Our data showed a seasonality to tick activity consistent with previous reports with peaks of activity at the start of the summer and minimal activity during winter [4, 23–25]. Although we are unable to identify the life cycle stage of the ticks referred to within the EHRs, we assume this seasonality largely reflects that of adult ticks, and to a lesser extent nymphs, of *I. ricinus,* as these are the most common ticks found on companion animals in GB [4, 6, 7, 24, 26]. This tick is susceptible to desiccation and its host-seeking behaviour (questing) is greatly influenced by changes in temperature and humidity [27]. It therefore has an annual variation of peak activity, with levels rising in early spring and peaking between April and July, with low levels in winter [24], although this may show regional variation [28]. The fact that the seasonal profile we observed in dogs was similar to that in the total population, is a reflection of the demographic predominance of dogs in our data and in other such veterinary visiting populations [29, 30].

Interestingly, tick activity on cats showed marked differences to that seen in dogs, raising several important questions relating both to ectoparasite biology, as well as owner and veterinary surgeon behaviour. Our study is the first to suggest that cats are more likely to present to veterinary clinics with ticks than dogs. Previous studies based on tick submissions either excluded cats [6, 7] or lacked suitable population denominator data to calculate a relative risk [4]. Whether this represents a genuine increased risk of ticks on cats, or that ticks on cats are more likely to be observed by owners and presented to the veterinary surgery, or whether veterinary surgeons are more likely to record ticks on cats in their EHRs, remains to be determined. As well as this overall increased risk, cats continued to present with ticks during the winter of 2015–2016, in contrast to dogs where there were short periods where ticks were not identified in this population. During these months, dog owners may be less inclined to take their dogs for exercise where they may be exposed to ticks due to shortened day length, cooler temperatures and higher rainfall. However, domestic cats in the UK may remain susceptible to ticks, albeit at lower levels, due to their ability to explore outside habitats at their own free will due to the common use of cat flaps. The indoor or outdoor nature of a cat is not explicitly recorded within the SAVSNET population, unless it has been recorded within the EHR. This potential risk factor was therefore not studied.

Ticks on cats also showed a different temporal pattern of tick activity with an earlier main peak in the spring and some evidence for a second smaller peak in the autumn. The precise reason for this apparent difference remains unknown but may relate to differences in host susceptibility to different tick species. Cats are significantly more likely to carry *Ixodes hexagonus* than *I. ricinus* [26] and *I. hexagonus* is more frequently found on cats than dogs [4, 5, 26]. The activity of *I. hexagonus* is closely linked to the density and behaviour of its primary host, the European hedgehog (*Erinaceus europaeus*) [31]. *I. hexagonus* is more prevalent earlier in the year than *I. ricinus* [31, 32], coinciding with the emergence of hedgehogs from hibernation [33], and possibly explaining the earlier peak of tick activity we identified in cats. The second autumnal peak could represent interaction between cats and hedgehogs at a time when hedgehogs are preparing for hibernation and juveniles are gaining independence, leading to greater hedgehog numbers being seen [33], all at a time when *I. hexagonus* is also at great abundance on the hedgehogs themselves [31, 32].

The spatial distribution we describe generally mirrors previous work on tick distributions in GB [4–7, 23]. Comparing it to the most recent study published using data from a shorter, but overlapping time period (16 weeks between April and July 2015), both studies identified the highest levels of tick activity in southern postcode areas of England, with high levels also in the south of Scotland [6]. However, in contrast, we see higher levels of activity in north and mid-Wales, and north-west England, and less clear areas of high activity in north Norfolk and the north-east of England. These observations likely reflect differences in methodology used by the two projects including veterinary clinic recruitment, the period of sampling and potentially tick distribution [6].

Collection of continuous surveillance data over 2 years across GB has allowed us to begin to describe a complex mosaic of tick activity across the country in different seasons. However, broad trends can be identified. In winter, low levels of tick activity remain throughout England and Wales, challenging the belief of some vets, who recorded in their EHRs that ticks pose no risk in winter (unpublished observations). The results also showed that the timing of peak activity varied by postcode area, with the majority of areas peaking in the spring, the remainder peaking in the summer. The dataset described here represents a rich research tool in which to explore the varied impact of climate, and other environmental and ecological factors, on tick activity.

To maintain a high specificity, we applied a very restrictive case definition, only including ticks that were seen by a veterinary surgeon or nurse and recorded during the consultation. Therefore, it is clear that not all ticks on cats and dogs will be included in our study. Many ticks on companion animals will not present to the veterinary practice either because the owner is not concerned, or removed the tick themselves, or the ticks were not noticed. Equally ticks on animals in a veterinary consultation may not be noticed, or not recorded, especially where they are incidental findings in relation to what may be a more serious clinical need. Indeed, where dogs had a bespoke thorough clinical examination as part of a research study to identify tick carriage, reported tick prevalence was much higher (30%) [6]. This study was however carried out during peak tick activity (April–July) and as the authors stated, practitioners participating in the study may have been more likely to sample animals with observed ticks on them. Although it is clear that the values we report are therefore an underestimate of overall tick activity on companion animals, we feel confident that they can describe relevant levels of relative risk. We must also acknowledge that health scares and media coverage could influence owner behaviour and veterinary recording behaviour. This has been previously discussed in relation to the *Babesia canis* outbreak seen in early 2016 [17]. However, in this particular case, this outbreak did not appear to influence the overall temporal trends of our data (data not presented).

Arrival of exotic ticks has been of great concern to both the veterinary and medical professions as they have the potential to carry pathogens not currently transmitted in the UK [34–37]. This has driven a need for species-level surveillance of ticks such as provided by PHE [5] and the Big Tick project [6]. Within our data, only five EHRs included information at the genus and species level; two referring to *Ixodes* spp, one to *I. ricinus*, one to *Dermacentor* spp and one EHR referring to both *Dermacentor and Rhipicephalus* spp. Although these numbers are clearly low and in the absence of microscopic confirmation need to be treated with some caution, they still raise important questions. Whilst a few foci of *Dermacentor* are known to exist in the UK [5], *Rhipicephalus sanguineus* has only been reported in dogs that have travelled in the rest of Europe [6, 37], such that reference to *Rhipicephalus* spp in even one EHR could be significant. The infrequent mention of tick species likely reflect time constraints of a short consultation, the challenge of identification, especially if the tick is engorged, and veterinary surgeons deeming it clinically irrelevant. In the future, the reference to rare and exotic tick species identified by EHR surveillance, could be followed up by submission of the tick to relevant health authorities with tick identification capabilities; such as PHE. Surveillance systems based on EHRs would be improved if veterinary surgeons were encouraged to record within the EHR any recent travel history and information about tick species where they are confident to do so.

The current limitations of this study are inherent to its methodology. Since recruitment of practices is not random, there may be selection bias in our results, meaning generalisability to the entire UK population of veterinary visiting dogs and cats is not possible. In addition, population statistics for companion animals in the UK are generally poor or unavailable, such that our results cannot be described by incidence; this may change as compulsory microchipping of dogs has recently come into legislation [38]. Some postcode areas have relatively small amounts of data and were excluded from our analyses. However, the fact that 56% (70 of 124) of postcode areas contributed more than 5000 EHRs during the study period, and that 42% (52 of 124) of areas contributed more than 10 000 EHRs suggests that we already have good data coverage for large parts of GB. As SAVSNET continues to expand through clinic recruitment, we believe that the spatial distribution of clinics and the number of EHRs collected will become more homogenous. Our data will always underestimate true tick activity on companion animals, and veterinary surgeons or nurses rarely record the tick species in EHR. In addition, like other studies that define a tick's location by the pet owner's postcode [6], our results should be seen as a proxy for tick activity at a given geographical area, rather than the location where the animal necessarily acquired the tick.

Despite these current limitations, we believe this form of surveillance offers some real benefits. Using EHRs is very passive in nature, as once a veterinary clinic has been enrolled, no changes in clinician behaviour need occur for the data to be captured. This data is collected in real-time, with the only rate-limiting step currently being the time taken to verify the strict case definition. Research is currently being undertaken to enhance the specificity of our free-text analysis approach by utilising natural language processing, this will improve the level of automation and reduce the number of cases to be verified. Compared with systems that rely on the general public identifying a tick, ticks recorded in EHRs are identified by a qualified health care professional [9]. There is also minimal labour required, except the upkeep of a system to collect the EHRs on which it relies. As our results are similar to previous surveillance and field work performed in the UK, we believe that this method provides a novel and complementary approach for tick surveillance that could be adopted by other countries where mature pet animal EHRs exist. As more clinics are recruited the representativeness of such systems can be improved. Linking data through postcode to other data sources, such as habitat type and localised meteorological data, will provide new opportunities to understand the effect of climate change and land use changes on the distribution and activity levels of ticks [34, 35, 39–41].

In summary, this study shows how the passive real-time collection of companion animal EHRs can provide efficient, accurate and novel data on tick activity in a large national sentinel population of companion animals. We highlight for the first time, temporal differences of tick exposure between domesticated cats and dogs. As the availability of EHRs increases, such methodology can provide a comprehensive temporal and spatial understanding of tick activity, and in combination with other systems already in place, has the potential to further inform tick and TBD risk models, aiding a ‘One-Health’ approach for public health messaging and tick control.

## ETHICS APPROVAL AND CONSENT TO PARTICIPATE

Ethics approval for this project came from the University of Liverpool research committee (RETH00964). Consent to participate is recognised via an opt out process available to all companion animal owners at veterinary clinics participating in SAVSNET.

## AVAILABILITY OF DATA AND MATERIALS

The datasets generated during and/or analysed during the current study are not publicly available due to issues of companion animal owner confidentiality, but are available on request from the SAVSNET Data Access and Publication Panel (savsnet@liverpool.ac.uk) for researchers who meet the criteria for access to confidential data.
